# Low‐intensity pulsed ultrasound alleviates doxorubicin‐induced cardiotoxicity via inhibition of S100a8/a9‐mediated cardiac recruitment of neutrophils

**DOI:** 10.1002/btm2.10570

**Published:** 2023-07-07

**Authors:** Hong Zhu, Min He, Yong‐Li Wang, Yuanxin Zhang, Jingsong Dong, Bo‐Yan Chen, Yu‐Lin Li, Lu‐Jun Zhou, Lin‐Juan Du, Yuan Liu, Wu‐Chang Zhang, Dean Ta, Sheng‐Zhong Duan

**Affiliations:** ^1^ Laboratory of Oral Microbiota and Systemic Diseases Shanghai Ninth People's Hospital, College of Stomatology, Shanghai Jiao Tong University School of Medicine Shanghai China; ^2^ Translational Medical Center for Stem Cell Therapy & Institutes for Regenerative Medicine, Shanghai East Hospital, Tongji University School of Medicine Shanghai China; ^3^ Center for Biomedical Engineering, School of Information Science and Technology, Fudan University Shanghai China; ^4^ National Center for Stomatology; National Clinical Research Center for Oral Diseases; Shanghai Key Laboratory of Stomatology Shanghai China; ^5^ Department of Cardiology Ninth People's Hospital, Shanghai Jiao Tong University School of Medicine Shanghai China; ^6^ Department of Rehabilitation Medicine Huashan Hospital, Fudan University Shanghai China

**Keywords:** cardiotoxicity, doxorubicin, LIPUS, neutrophil chemotaxis, S100a8/a9

## Abstract

Doxorubicin (DOX)‐induced cardiotoxicity limits its broad use as a chemotherapy agent. The development of effective and non‐invasive strategies to prevent DOX‐associated adverse cardiac events is urgently needed. We aimed to examine whether and how low‐intensity pulsed ultrasound (LIPUS) plays a protective role in DOX‐induced cardiotoxicity. Male C57BL/6J mice were used to establish models of both acute and chronic DOX‐induced cardiomyopathy. Non‐invasive LIPUS therapy was conducted for four consecutive days after DOX administration. Cardiac contractile function was evaluated by echocardiography. Myocardial apoptosis, oxidative stress, and fibrosis were analyzed using terminal deoxynucleotidyl transferase‐mediated dUTP nick end labelling (TUNEL) staining, dihydroethidium (DHE) staining, and picrosirius red staining assays. RNA‐seq analysis was performed to unbiasedly explore the possible downstream regulatory mechanisms. Neutrophil recruitment and infiltration in the heart were analyzed by flow cytometry. The S100a8/a9 inhibitor ABR‐238901 was utilized to identify the effect of S100a8/a9 signaling. We found that LIPUS therapy elicited a great benefit on DOX‐induced heart contractile dysfunction in both acute and chronic DOX models. Chronic DOX administration increased serum creatine kinase and lactate dehydrogenase levels, as well as myocardial apoptosis, all of which were significantly mitigated by LIPUS. In addition, LIPUS treatment prevented chronic DOX‐induced cardiac oxidative stress and fibrosis. RNA‐seq analysis revealed that LIPUS treatment partially reversed alterations of gene expression induced by DOX. Gene ontology (GO) analysis of the downregulated genes between DOX‐LIPUS and DOX‐Sham groups indicated that inhibition of neutrophil chemotaxis might be involved in the protective effects of LIPUS therapy. Flow cytometry analysis illustrated the inhibitory effects of LIPUS on DOX‐induced neutrophil recruitment and infiltration in the heart. Moreover, S100 calcium binding protein A8/A9 (S100a8/a9) was identified as a potential key target of LIPUS therapy. S100a8/a9 inhibition by ABR‐238901 showed a similar heart protective effect against DOX‐induced cardiomyopathy to LIPUS treatment. LIPUS therapy prevents DOX‐induced cardiotoxicity through inhibition of S100a8/a9‐mediated neutrophil recruitment to the heart, suggesting its potential application in cancer patients undergoing chemotherapy with DOX.

**Abbreviations**
ABR: ABR‐238901
CAT: catalase
CK: creatine kinase
DEG: differentially expressed genes
DHE: dihydroethidium
DOX: doxorubicin
GO: gene ontology
IL‐1β: interleukin‐1β
LDH: lactate dehydrogenase
LIPUS: low‐intensity pulsed ultrasound
LVEF: left ventricular ejection fraction
LVFS: left ventricular fractional shortening
MDA: malonaldehyde
MPO: myeloperoxidase
NLRP3: NLR family pyrin domain‐containing 3
PBS: phosphate buffer saline
S100a8: S100 calcium binding protein A8
S100a9: S100 calcium binding protein A9
TUNEL: terminal deoxynucleotidyl transferase‐mediated dUTP nick end labelling

## 1 BACKGROUND

Doxorubicin (DOX), an anthracycline agent, is widely used to treat tumor patients because of its broad‐spectrum antitumor activity.^1^, ^2^ However, its high affinity to cardiomyocytes results in progressive dilated cardiomyopathy in a cumulative dose‐dependent way, which greatly limits its therapeutic effects.^1^, ^3^ Therefore, effective intervention strategies are urgently needed to reduce the cardiac side effects of DOX. Mechanistically, DOX induces cardiomyopathy through local cytotoxicity and systemic immune responses. On one hand, the local cytotoxic mechanism involves DNA damage, mitochondrial dysfunction, oxidative stress, inflammation, and cardiomyocyte death.^4^, ^5^ On the other hand, DOX‐elicited systemic immune responses, including neutrophil chemotaxis and cardiac recruitment, are increasingly recognized as important mechanisms that drive the progression of DOX‐induced cardiomyopathy.^6^, ^7^

Low‐intensity pulsed ultrasound (LIPUS) is a low‐power ultrasonic pulse with a certain repetition frequency, which can transmit mechanical energy into biological tissues.^8^ Through changing micromechanical stress in local tissues, LIPUS causes a series of biological reactions to accelerate the repair process of tissue injuries.^8^, ^9^ Notably, LIPUS has shown great therapeutic potential in cardiovascular diseases such as myocardial infarction,^10^ cardiac fibrosis,^11^, ^12^ viral myocarditis,^13^ and heart failure with preserved left ventricular ejection fraction.^14^ The molecular mechanisms of LIPUS therapy mainly involve regulations of oxidative stress,^12^ inflammation^11^, ^12^, ^13^ and endothelial function.^10^, ^14^ Interestingly, a recent study has demonstrated that brief exposure to LIPUS was sufficient to eliminate paclitaxel treatment‐induced cytotoxicity, and might provide a new strategy to counter chemotherapy‐induced peripheral neuropathy and alopecia.^15^ However, it remains unknown whether and how LIPUS therapy affects DOX‐induced cardiotoxicity.

In the present study, we aimed to investigate the therapeutic effects of LIPUS on DOX‐induced cardiotoxicity and explore the underlying mechanisms. We first established mouse models of acute and chronic DOX‐induced cardiotoxicity and examined the effect of LIPUS treatment on DOX‐induced heart contractile dysfunction, myocardial injury, apoptosis, cardiac oxidative stress, and fibrosis. Then we used RNA‐seq analysis to unbiasedly explore the downstream signaling and molecular targets and revealed that inhibition of S100a8/a9‐mediated neutrophil chemotaxis might be involved in the beneficial effects of LIPUS. We further examined the role of ABR‐238901, a potent S100a8/a9 inhibitor, in acute DOX‐induced cardiotoxicity. Our study illustrated the beneficial role of non‐invasive LIPUS in preventing DOX‐induced cardiotoxicity, possibly through inhibition of S100a8/a9‐mediated cardiac neutrophil recruitment.

## 2 MATERIALS AND METHODS

### 2.1 Animal experiments

C57BL/6J wild‐type male mice (5 weeks old) were purchased from Gempharmatech Inc (Jiangsu, China). Mice were housed in a temperature‐controlled (22 ± 1°C) and relative humidity‐controlled (50% ± 5%) environment with a 12 h dark–light cycle, given a standard chow diet and drinking water ad libitum. All animal experiments were performed by the approval of the Institutional Animal Care and Use Committee of the Institute of developmental biology of Fudan University (approval number: IDM2021046). Ten weeks old mice were used for later animal experiments and were in accordance with the Animal Research: Reporting of In Vivo Experiments (ARRIVE) guidelines 2.0. All mice were randomly assigned to different groups following simple randomization procedures through a computerized random number generator. For the acute DOX experiment, 29 mice were randomly assigned to four groups: Control‐Sham group (*n* = 7), Control‐LIPUS group (*n* = 7), DOX‐Sham group (*n* = 7), and DOX‐LIPUS group (*n* = 8). Mice in DOX‐Sham and DOX‐LIPUS groups were intraperitoneally injected with one dose of 18 mg/kg DOX (D1515, Sigma) as previously described.^16^ Mice in the control groups were given the same dose of saline. The mice were then treated with LIPUS or sham operation for four consecutive days. On day 6, cardiac function was measured by transthoracic echocardiography and the mice were sacrificed. For the chronic DOX experiment, 36 mice were randomly assigned to three groups: Control‐Sham group (*n* = 11), DOX‐Sham group (*n* = 12), and DOX‐LIPUS group (*n* = 13). Mice in DOX‐Sham and DOX‐LIPUS groups were intraperitoneally injected with 6 mg/kg DOX once a week for three times (18 mg/kg in total) as previously described.^17^ LIPUS or sham operation was conducted for four consecutive days after each DOX injection, and the mice were sacrificed 3 weeks after the start of the experiments. For S100a8/a9 inhibition experiment, 30 mg/kg ABR‐238901 (HY‐141537, MCE) was administered by oral gavage for three consecutive days^18^, ^19^ after one dose of 18 mg/kg DOX. On day 6, cardiac function was measured and the mice were sacrificed.

### 2.2 LIPUS therapy

LIPUS devices were designed and manufactured by the Intelligent Medical Ultrasound Lab of Fudan University (Shanghai, China) as reported before.^20^, ^21^ Ultrasound parameters, including frequency, intensity, duty cycle, and treatment duration, are modifiable by software applications. Based on our previous studies,^20^, ^21^ the LIPUS therapy was conducted under the following conditions: frequency 1.0 MHz, duty cycle 20%, voltage applied on transducer 28.592 volts (V), *I*_sata_ (spatial average temporal average) 110 mW/cm^2^, and duration 15 min. Before LIPUS treatment, mice were anesthetized with isoflurane and hairs were removed from the chest of mice using hair removal creams. To have a stable sound field, ultrasonic coupling agent (Hynaut, Shandong, China) was coated on the mouse chest and the transducer. A plane transducer with 18 mm diameter, which covers the whole heart of a mouse, was fixed on a holder to ensure that the treatment location was kept unchanged during the whole treatment period. The mice in the Sham groups were undergone the same procedures including anesthesia without LIPUS therapy.

### 2.3 Echocardiography analysis

Transthoracic echocardiography was performed to evaluate cardiac function. Mice were placed on a platform and lightly anesthetized with 1.5% isoflurane inhalation. Heart rate during the echocardiographic study was maintained in the range of 500–550 bpm for M‐mode and B‐mode. Echocardiography was conducted to obtain echocardiographic parameters using Vevo 3100LT (Visual Sonics, Ontario, Canada), including left ventricular ejection fraction (EF%) and left ventricular fractional shortening (FS%).

### 2.4 Serum biochemical analysis

Blood samples were collected from mice and sera were obtained by centrifugation at 3500 rpm for 15 min at 4°C. Serum lactate dehydrogenase (LDH), creatine kinase (CK) levels, catalase (CAT), and malonaldehyde (MDA) were measured using colorimetric assay kits according to the manufacturer's instructions (Nanjing Jiancheng Bioengineering Institute, Nanjing, China). Serum interleukin‐1β (IL‐1β) was measured using a mouse ELISA assay kit (MEC1010, Anogen).

### 2.5 Histology and morphometric analysis

Hearts were quickly removed from mice, washed with phosphate buffer saline (PBS), fixed in 4% paraformaldehyde for 24 h, and embedded in paraffin. Cardiac sections (5‐μm) were prepared and stained with picrosirius red to evaluate collagen deposition. For each section, at least five random fields were analyzed to determine the percentage of fibrosis. Interstitial fibrosis was quantified as picrosirius red‐positive area/total area and perivascular fibrosis was quantified as perivascular collagen area/lumen area. Data were quantified using Image J software (National Institutes of Health).

### 2.6 Immunofluorescence staining

Paraffin sections of hearts were deparaffinized and rehydrated, then incubated in blocking buffer at room temperature for 1 h after antigen retrieval treatment. The sections were then incubated with primary antibodies and fluorochrome‐conjugated secondary antibodies sequentially. Finally, the sections were counterstained with DAPI. To assess myocardial apoptosis, terminal deoxynucleotidyl transferase‐mediated dUTP nick end labelling (TUNEL) staining was performed. TUNEL‐positive cells were counted from at least five different microscopic fields of each section. The percentage of TUNEL‐positive cardiac cells was calculated and expressed as % of total nuclei identified by DAPI staining. The following primary antibodies were used: rabbit anti‐S100a8/a9 (abcam, ab288715), mouse anti‐myeloperoxidase (MPO) (Servicebio, GB12224), rabbit anti‐MPO (abcam, ab208670).

### 2.7 Dihydroethidium (DHE) staining

After removal from mice, heart tissues were washed with PBS and embedded in optimal cutting temperature compound (Sakaru, Japan). Frozen sections with 7‐μm thickness were prepared, washed in PBS for 5 min, and incubated with 2 × 10^−6^ M DHE (D11347, Life Technologies) in the dark at 37°C for 30 min. At least 5 different microscopic fields of each section were photographed. DHE fluorescent intensity was analyzed by a blinded investigator using Image J software.

### 2.8 Flow cytometry

Heart tissues were minced and placed in a digestion solution containing 1.5 mg/mL Collagenase II (Worthington, Lakewood, NJ, USA), 1.5 mg/mL Collagenase IV (Worthington), and 60 U/mL DNase I (AppliChem, Lochem, Darmstadt, Germany) in hank's balanced salt solution. The samples were first dissociated mechanically with a gentle magnetic activated cell sorting Dissociator system (Miltenyi Biotec, Bergisch Gladbach, Germany), and then digested at 37°C for 20 min. Single‐cell suspensions were obtained by filtering digested tissues through 70‐μm cell strainers (BD Biosciences, San Jose, CA, USA). Blood was collected in EDTA‐coated tubes. For both hearts and blood, red blood cells were lysed before staining. Single‐cell suspensions were centrifuged, blocked with Fc block for 10 min, and labeled with antibodies at 4°C for 20 min. The following antibodies were used: CD45‐APC‐CY7, CD11b‐FITC, Ly6G‐BV711, and Ly6C‐PE‐Cy7. All samples were analyzed using LSR Fortessa (BD Biosciences).

### 2.9 RNA sequencing

Heart tissues were preserved in RNAlater and sent to OE Biotech Co., Ltd. (Shanghai, China) for sequencing. Total RNA was extracted using the TRIzol reagent according to the manufacturer's protocol. RNA purity and quantification were evaluated using a NanoDrop 2000 spectrophotometer (Thermo Scientific, USA). RNA integrity was assessed using an Agilent 2100 Bioanalyzer (Agilent Technologies, Santa Clara, CA, USA). A sequencing library was constructed using TruSeq Stranded mRNA LT Sample Prep Kit (Illumina, San Diego, CA, USA) according to the manufacturer's instructions. Sequencing was carried out on an Illumina HiSeq X Ten platform and 150 bp paired‐end reads were generated. Differentially expressed genes (DEGs) were identified by >1.5‐fold change in gene expression with *p*‐value <0.05. Z‐score normalization was executed for heatmap visualization of DEGs. GO enrichment analysis of DEGs was performed using R based on the hypergeometric distribution.

### 2.10 Quantitative real‐time PCR

Total RNA from tissues was extracted using TRIzol (Thermo Fisher Scientific), and cDNA was synthesized using reverse transcription kits (RR037A, Takara) according to the manufacturer's instructions. Quantitative PCR was performed using SYBR Green Mix (Takara, Japan) on a Light Cycler 480II (Roche). Relative gene expression was determined by normalizing to GAPDH. Primer sequences are listed in Table S1.

### 2.11 Statistical analysis

Data analyses were performed using Prism 8.3 (GraphPad Software, San Diego, USA). Results were shown as mean ± standard error of mean (SEM) for all experiments. The normality of data were assessed using the Shapiro–Wilk test. For normally distributed data, unpaired two‐tailed Student's *t*‐test was performed to compare the differences between two groups, and one‐way analysis of variance (ANOVA) or two‐way ANOVA followed by Tukey's post‐hoc test was performed to compare multiple groups. For non‐normal variables, the Mann–Whitney *U* test was used to compare two groups, and the Kruskal–Wallis test followed by Dunn's multiple comparison test was performed to compare more than two groups. The *p* values <0.05 were considered statistically significant.

## 3 RESULTS

### 3.1 LIPUS protects against DOX‐induced cardiac dysfunction, myocardial injury, and apoptosis

**FIGURE 1:**
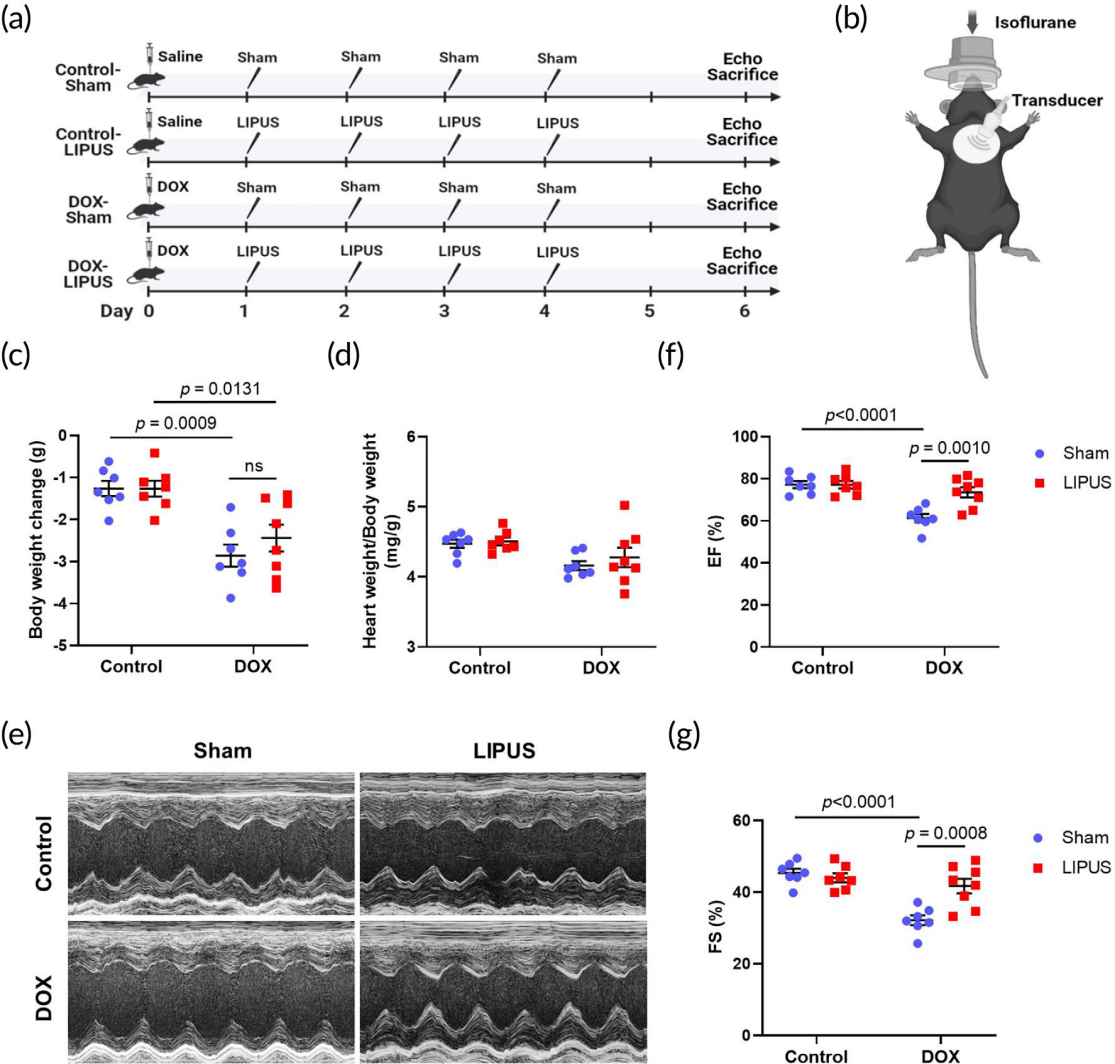
LIPUS protects against acute DOX‐induced cardiac dysfunction. (a) Experimental design is shown in the schematic overview. Male wild‐type C57BL6/J mice were used for the experiments. (b) Schematic illustration of LIPUS treatment. (c) Quantifications of body weight changes of mice after DOX administration. *n* = 7:7:7:8. (d) Quantifications of heart height/body weight of mice. *n* = 7:7:7:8. (e) Representative M‐mode echocardiographic images of mice. (f, g) Quantifications of ejection fraction (EF) and fractional shortening (FS) based on echocardiography. *n* = 7:7:7:8. Values are expressed as mean ± SEM. Data were analyzed using two‐way ANOVA with Tukey's test for multiple comparisons.

To assess the therapeutic effect of LIPUS treatment on DOX‐induced cardiotoxicity, we first established an acute DOX injury model with one injection of 18 mg/kg DOX, then treated the mice with LIPUS or sham operation for four consecutive days. On day 6, cardiac function was measured by transthoracic echocardiography and then the mice were sacrificed (Figure 1a,b). Acute DOX administration caused a significant decrease in body weights in both DOX‐Sham and DOX‐LIPUS groups compared with the control groups, while no significant difference was found between the DOX‐Sham group and DOX‐LIPUS group (Figure 1c). Mice in both DOX‐Sham and DOX‐LIPUS groups tended to have lower heart weight/body weight ratios compared to those in the control groups, although the differences did not reach statistical significance (Figure 1d). Notably, echocardiographic results showed that acute DOX administration caused a significant decrease in left ventricular EF and FS in the DOX‐Sham group compared to the Control‐Sham group, indicating deterioration of cardiac systolic function, while LIPUS treatment almost completely reversed the deterioration in the DOX‐LIPUS group compared to the DOX‐Sham group (Figure 1e–g).

**FIGURE 2:**
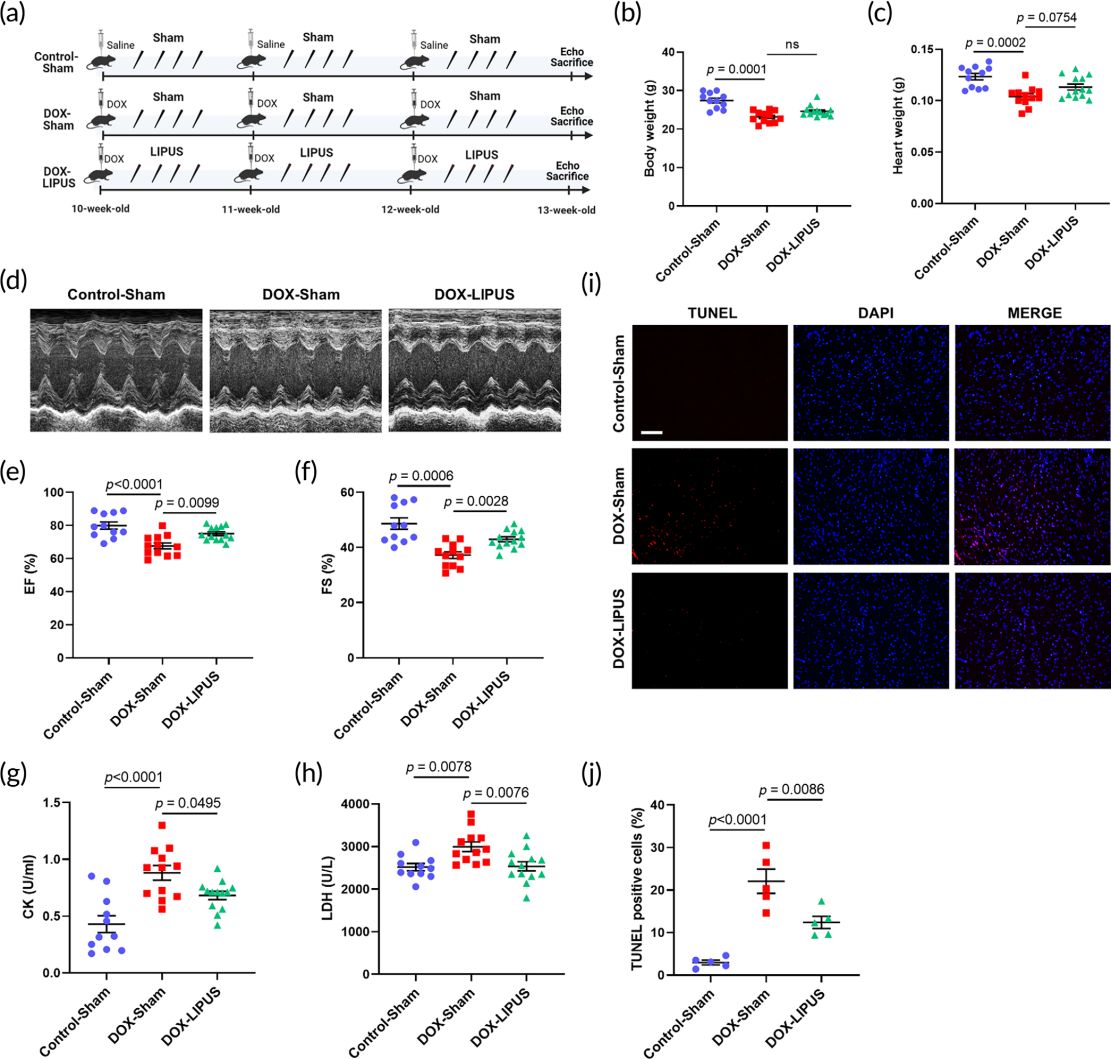
LIPUS alleviates chronic DOX‐induced cardiac dysfunction, myocardial injury, and apoptosis. (a) Experimental design is shown in the schematic overview. Male wild‐type C57BL6/J mice were used for the experiments. (b) Quantifications of body weights of mice after DOX administration. *n* = 11:12:13. (c) Quantifications of heart heights of mice. *n* = 11:12:13. (d) Representative M‐mode echocardiographic images of mice. (e, f) Quantifications of ejection fraction (EF) and fractional shortening (FS) based on echocardiography. *n* = 11:12:13. (g, h) Quantifications of serum creatine kinase (CK) and lactate dehydrogenase (LDH) in mice with different treatments. *n* = 11:12:13. (i) Representative images of TUNEL staining of mouse cardiac sections. Scale bar: 100 μm. (j) Quantifications of the percentages of apoptotic cells in the heart based on TUNEL staining. *n* = 5:5:5. Values are expressed as mean ± SEM. Data were analyzed using one‐way ANOVA with Tukey's test for multiple comparisons (c, e, f, g, h, j) or Kruskal–Wallis test with Dunn's multiple comparisons test (b). ns indicates not significant.

Since DOX‐associated cardiotoxicity is usually caused by chronic DOX treatment in clinical settings, we then established a chronic DOX injury model with three injections of a total of 18 mg/kg DOX. LIPUS or sham was conducted for four consecutive days after each DOX injection, and the mice were sacrificed 3 weeks after the start of the experiments (Figure 2a). Chronic DOX administration caused a significant decrease in both body weights and heart weights, while LIPUS tended to increase both parameters in the DOX‐LIPUS group (Figure 2b,c). Echocardiographic analyses showed that chronic DOX administration significantly decreased both left ventricular EF and FS, while LIPUS treatment partially reversed the reduction, leading to markedly higher EF and FS in the DOX‐LIPUS group than in the DOX‐Sham group (Figure 2d–f). In addition, serum levels of CK and LDH, which are indicators of cardiac injury, were significantly increased after chronic DOX administration; LIPUS therapy significantly lowered them in the DOX‐LIPUS group compared to the DOX‐Sham group (Figure 2g,h). TUNEL staining showed that chronic DOX administration significantly increased apoptotic myocardial cells, while LIPUS treatment significantly decreased myocardial apoptosis in the DOX‐LIPUS group (Figure 2i,j). These results together demonstrated the effectiveness of LIPUS therapy in improving DOX‐induced cardiotoxicity.

### 3.2 LIPUS alleviates DOX‐induced cardiac oxidative stress and fibrosis

**FIGURE 3:**
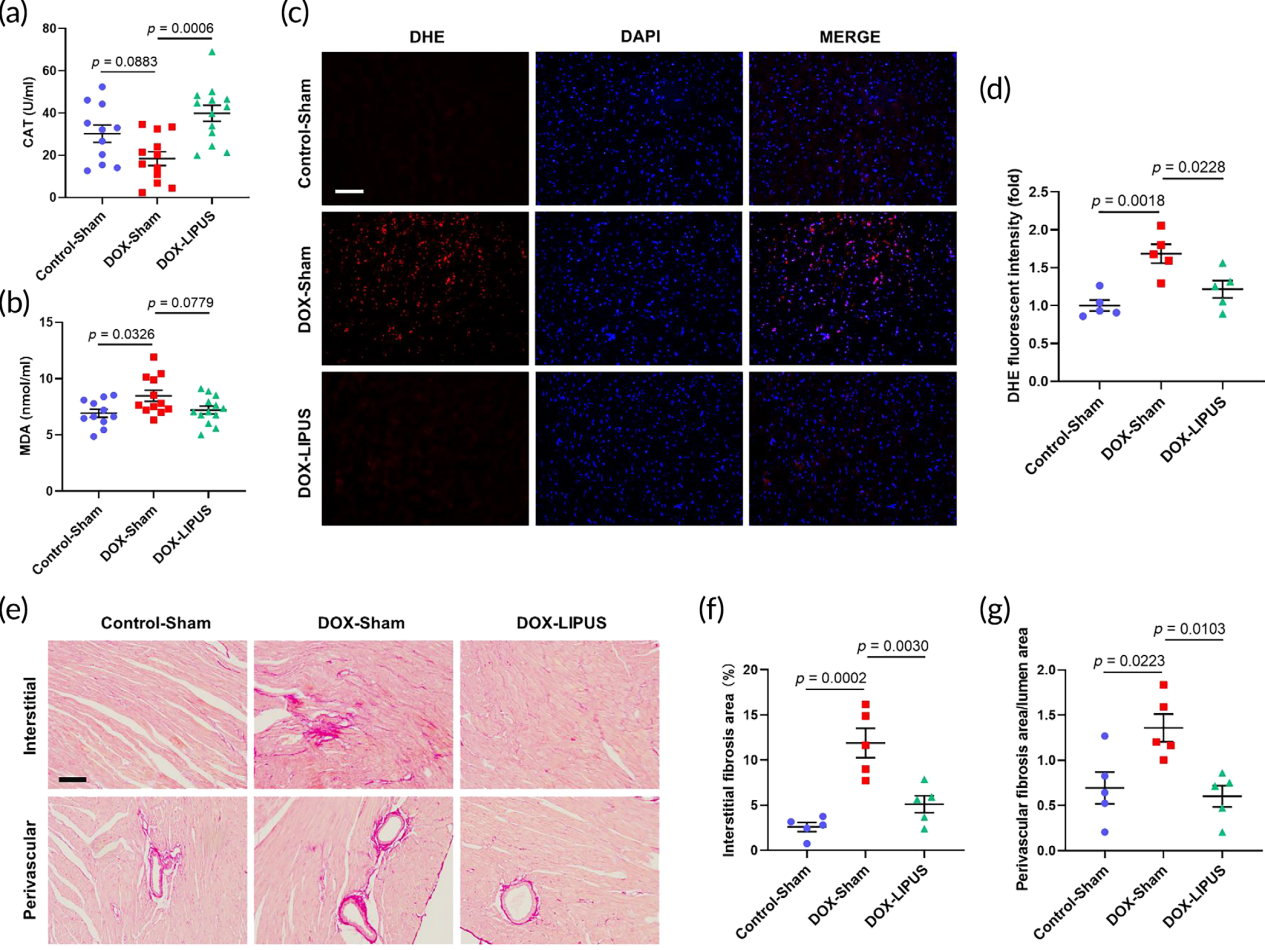
LIPUS alleviates DOX‐induced cardiac oxidative stress and fibrosis. (a, b) Quantifications of serum catalase (CAT) and malonaldehyde (MDA) in mice with different treatments. *n* = 11:12:13. (c) Representative images of DHE staining of mouse cardiac sections. Scale bar: 100 μm. (d) Quantifications of reactive oxygen species in the heart based on DHE staining. *n* = 5:5:5. (e) Representative images of Picrosirius red staining of mouse cardiac sections detecting interstitial and perivascular fibrosis. Scale bar: 100 μm. (f, g) Quantifications of interstitial fibrosis area/total area and perivascular fibrosis area/lumen area. *n* = 5:5:5. Values are expressed as mean ± SEM. Data were analyzed using one‐way ANOVA with Tukey's test for multiple comparisons.

Oxidative stress and cardiac fibrosis are typical pathological characteristics of DOX‐related cardiomyopathy.^4^, ^22^ Serum CAT level decreased and MDA level increased in the DOX‐Sham group compared with the Control‐Sham group, while LIPUS treatment significantly increased serum CAT and tended to decrease serum MDA in the DOX‐LIPUS group (Figure 3a,b). Additionally, DOX caused a significant increase in DHE immunofluorescent intensity in cardiac tissue, which was almost completely reversed by LIPUS treatment in the DOX‐LIPUS group (Figure 3c,d). Picrosirius red staining revealed significantly increased fibrosis in both interstitial and perivascular areas, which were substantially reduced in the DOX‐LIPUS group (Figure 3e,g). These results demonstrated that LIPUS therapy improved DOX‐induced cardiac oxidative stress and fibrosis.

### 3.3 LIPUS treatment inhibits neutrophil chemotaxis in DOX‐induced cardiotoxicity

**FIGURE 4:**
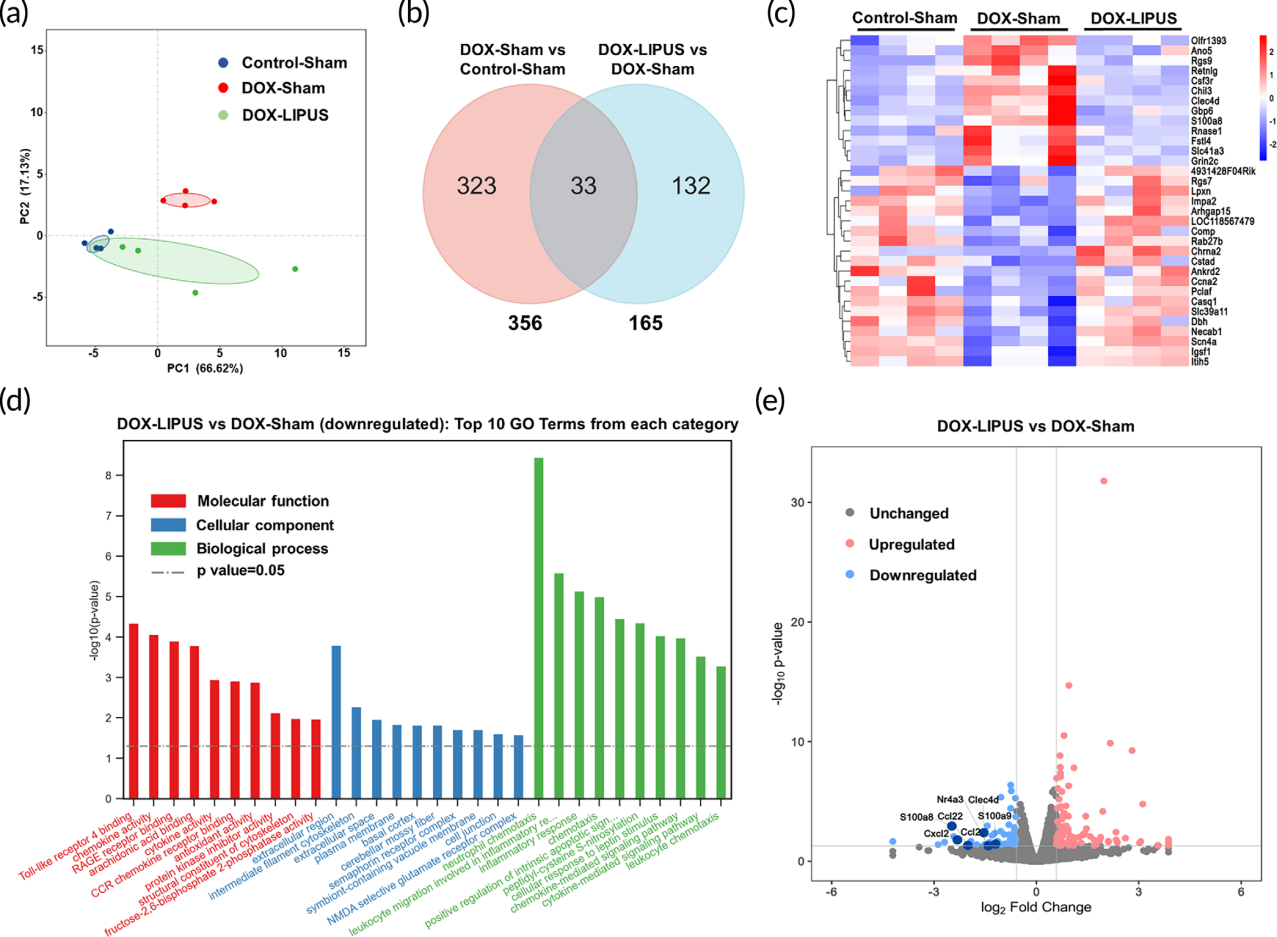
LIPUS treatment inhibits neutrophil chemotaxis in DOX‐induced cardiotoxicity. (a) Principal component analysis of RNA‐seq data of heart samples. *n* = 4:4:4. (b) Venn diagram presenting the overlap of differentially expressed genes (DEGs) between DOX‐Sham vs Control‐Sham and DOX‐LIPUS vs DOX‐Sham. DEGs are identified as fold change >1.5 and *p* < 0.05. (c) Heat map showing the overlapped DEGs in (b). The color bar represents the log‐transformed relative expression of genes. (d) Gene ontology (GO) analysis showing the top 10 terms from each of the three categories (molecular function, cellular component, and biological process) based on the downregulated DEGs between DOX‐LIPUS and DOX‐Sham. (e) Volcano plot of DEGs between DOX‐LIPUS and DOX‐Sham. Representative genes involved in neutrophil chemotaxis (S100a8, S100a9, Ccl2, Ccl22, Cxcl2) and inflammation (Nr4a3, Clec4d) are indicated.

RNA‐seq analysis was performed in cardiac tissues to obtain mechanistic insights into the molecular events involved in the protective effect of LIPUS therapy on chronic DOX‐induced cardiac injury. Principal component analysis of RNA‐seq data showed distinct clusters between the DOX‐Sham and Control‐Sham groups, as well as between DOX‐LIPUS and DOX‐Sham groups (Figure 4a). We identified 356 differentially expressed genes (DEGs) in the DOX‐Sham group compared to the Control‐Sham group, and 165 DEGs in the DOX‐LIPUS group compared to the DOX‐Sham group, and 33 overlapped DEGs shared by both comparisons (Figure 4b). The 33 overlapped DEGs were further displayed in heatmap (Figure 4c). Gene ontology (GO) analysis of the downregulated DEGs between DOX‐LIPUS and DOX‐Sham groups revealed that the top terms of molecular function and biological process were mostly associated with chemotaxis and inflammatory response, with neutrophil chemotaxis being the top‐ranked biological process, indicating that inhibition of neutrophil chemotaxis might be involved in the protective effect of LIPUS therapy (Figure 4d). The top terms in GO analysis of the upregulated DEGs did not seem to be relevant to DOX‐induced cardiotoxicity (Figure S1). The volcano plot of DEGs illustrated several downregulated genes involved in neutrophil chemotaxis, including S100a8, S100a9, Ccl2, Ccl22, and Cxcl2, as well as those involved in inflammation such as Nr4a3 and Clec4d (Figure 4e).

### 3.4 LIPUS inhibits cardiac recruitment of neutrophils in DOX‐treated mice

**FIGURE 5:**
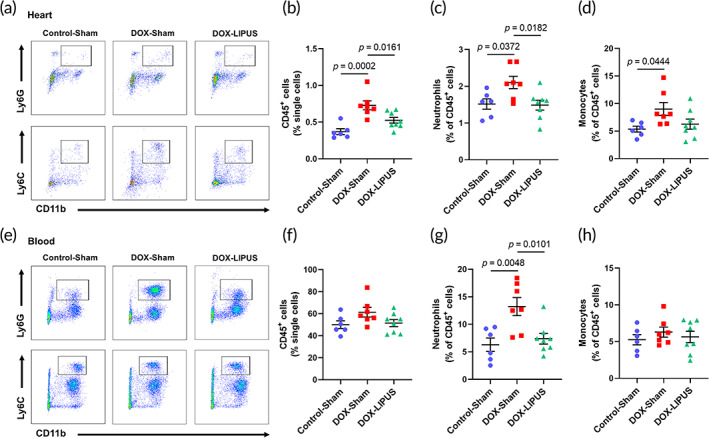
LIPUS inhibits cardiac recruitment of neutrophils in DOX‐treated mice. Representative flow cytometric analysis of CD11b^+^Ly6G^+^ neutrophils and CD11b^+^Ly6C^+^ monocytes in mouse hearts. (b–d) Quantification of CD45^+^ cells, CD11b^+^Ly6G^+^ neutrophils, and CD11b^+^Ly6C^+^ monocytes in mouse hearts. *n* = 6:7:8. (e) Representative flow cytometric analysis of CD11b^+^Ly6G^+^ neutrophils and CD11b^+^Ly6C^+^ monocytes in mouse blood. (f–h) Quantification of CD45^+^ cells, CD11b^+^Ly6G^+^ neutrophils, and CD11b^+^Ly6C^+^ monocytes in mouse blood. *n* = 6:7:8. Values are expressed as mean ± SEM. Data were analyzed using one‐way ANOVA with Tukey's test for multiple comparisons.

To further test whether neutrophil chemotaxis was involved in chronic DOX‐induced cardiac injury and the protective effect of LIPUS treatment, we performed flow cytometry to quantify the percentage of leukocytes, neutrophils and monocytes in both heart and blood (Figure S2). The results revealed that chronic DOX administration significantly increased the percentage of CD45^+^ leukocytes, CD11b^+^Ly6G^+^ neutrophils, and CD11b^+^Ly6C^+^ monocytes in the myocardium (Figure 5a–d). LIPUS treatment significantly inhibited the infiltration of leukocytes and neutrophils in the heart and showed a trend to decrease monocytes in the DOX‐LIPUS group (Figure 5a–d). In the blood, the percentage of neutrophils was significantly increased in DOX‐treated mice, which was substantially decreased by LIPUS treatment, but neither DOX nor LIPUS significantly affected the percentage of leukocytes or monocytes (Figure 5e–h). These results suggested that LIPUS therapy prevented DOX‐induced neutrophil recruitment to the heart.

### 3.5 LIPUS treatment mitigates S100a8/a9‐mediated neutrophil recruitment to the heart

**FIGURE 6:**
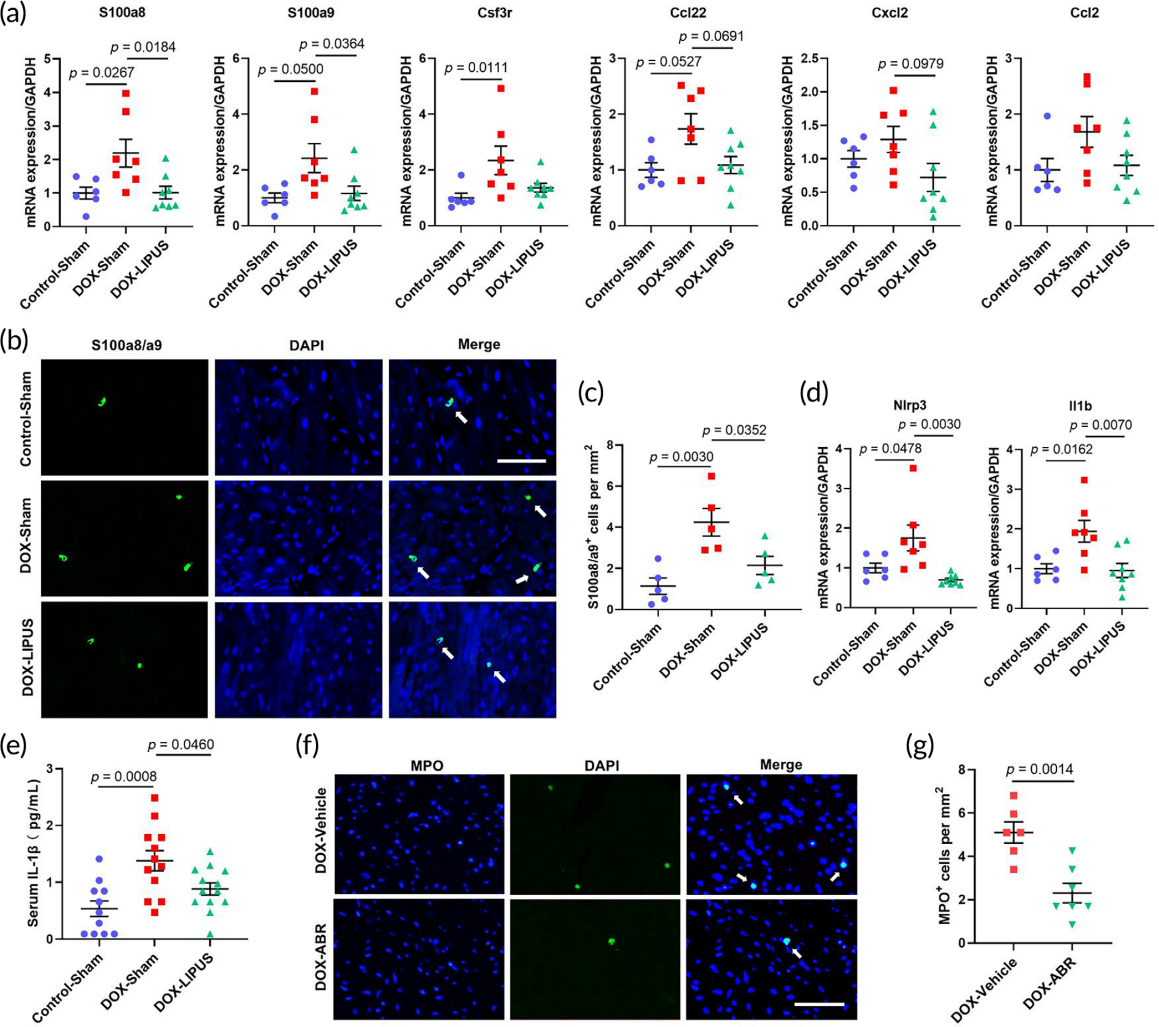
LIPUS treatment mitigates S100a8/a9‐mediated cardiac recruitment of neutrophils. (a) Quantitative RT‐PCR analysis of genes related to neutrophil chemotaxis in mouse heart samples. GAPDH was used for normalization. *n* = 6:7:8. (b) Representative immunofluorescence staining of S100a8/a9 in mouse cardiac sections. The arrows point to S100a8/a9‐positive cells. Scale bar: 100 μm. (c) Quantification of S100a8/a9^+^ cells/mm^2^ in the heart. *n* = 5:5:5. (d) Quantitative RT‐PCR analysis of *Il1b* and *Nlrp3* expression in mouse heart samples. *n* = 6:7:8. (e) Serum IL‐1β concentration in mice with different treatments. *n* = 11:12:13. (f) Representative immunofluorescence staining of MPO in mouse cardiac sections. The arrows point to MPO‐positive cells. Scale bar: 100 μm. (g) Quantification of MPO^+^ cells/mm^2^ in the heart. *n* = 6:7. Values are expressed as mean ± SEM. Data were analyzed using one‐way ANOVA with Tukey's test for multiple comparisons (a, c–e) or unpaired Student's *t*‐test (g).

To further explore the role of neutrophil chemotaxis in DOX‐induced cardiotoxicity and the protective effect of LIPUS therapy, we first used QRT‐PCR analysis to verify the DEGs associated with neutrophil chemotaxis detected by RNA‐seq. The results showed that the expression of both S100a8 and S100a9 significantly increased after chronic DOX administration, and were reversed by LIPUS treatment in the DOX‐LIPUS group (Figure 6a). The expression of the other genes related to neutrophil chemotaxis, including Csf3r, Ccl22, Cxcl2, and Ccl2, did not show a statistically significant difference between the DOX‐Sham and DOX‐LIPUS group (Figure 6a). Immunofluorescence staining of S100a8/a9 heterodimer showed that LIPUS therapy mitigated DOX‐induced increase of S100a8/a9‐positive cells in the heart (Figure 6b,c). S100a8/a9 is constitutively expressed in myeloid cells, especially neutrophils, but its expression can be induced in non‐myeloid cell types in the heart upon stimulations such as myocardial infarction and angiotensin‐II infusion.^23^, ^24^ Double immunofluorescence staining of S100a8/a9 and MPO (neutrophil marker) in DOX‐treated heart showed that S100a8/a9 was mostly colocalized with MPO (Figure S3), indicating that neutrophils being the main source of S100a8/a9. NLR family pyrin domain‐containing 3 (NLRP3)/IL‐1β signaling axis has been demonstrated to mediate S100a8/a9‐induced myelopoiesis in heart disease.^25^ QRT‐PCR analysis showed a significant increase in both Nlrp3 and Il‐1β mRNA expression after DOX administration, which were downregulated by LIPUS treatment in the DOX‐LIPUS group (Figure 6d). Moreover, results of ELISA showed that the serum level of IL‐1β was significantly higher after DOX administration, which was reversed by LIPUS therapy (Figure 6e). Then we used ABR‐238901, a potent S100a8/a9 inhibitor, to further test the essential role of S100a8/a9 in DOX‐induced neutrophil accumulation in the heart. The results showed that 3 days of ABR‐238901 administration significantly decreased the number of MPO‐positive neutrophils in the heart of mice treated with one dose of 18 mg/kg DOX (Figure 6f,g). These results revealed that the cardioprotective effect of LIPUS on DOX‐induced cardiotoxicity might be attributed to the inhibition of S100a8/a9‐mediated neutrophil recruitment to the heart.

### 3.6 S100a8/a9 inhibition by ABR‐238901 protects against DOX‐induced cardiotoxicity

**FIGURE 7:**
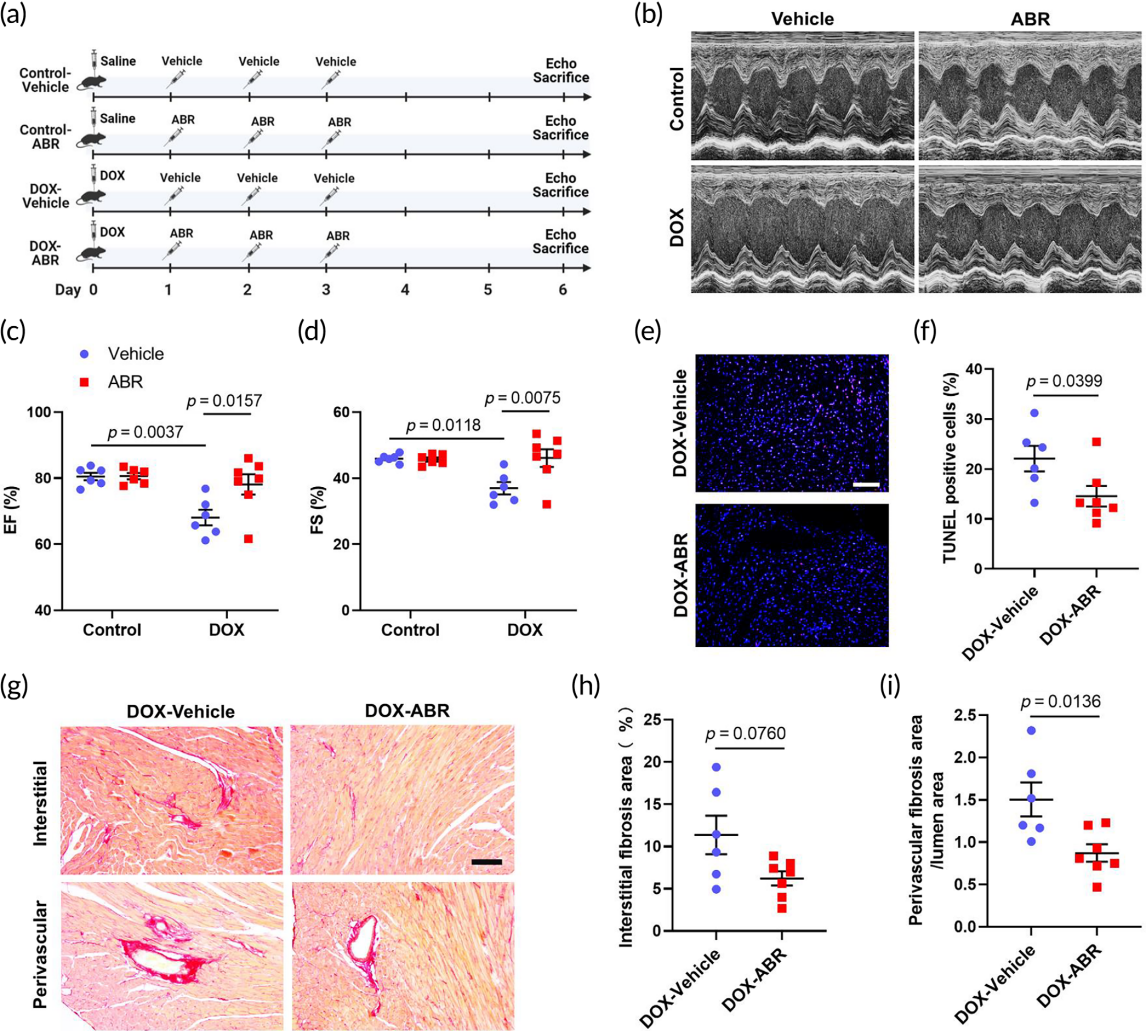
S100a8/a9 inhibition by ABR‐238901 protects against DOX‐induced cardiotoxicity. Experimental design is shown in the schematic overview. Male wild‐type C57BL6/J mice were used for the experiments. ABR: ABR‐238901. (b) Representative M‐mode echocardiographic images of mice with different treatments. (c, d) Quantifications of ejection fraction (EF) and fractional shortening (FS) based on echocardiography. *n* = 6:6:6:7. (e) Representative images of TUNEL staining of mouse cardiac sections. Scale bar: 100 μm. (f) Quantifications of the percentages of apoptotic cells in the heart based on TUNEL staining. *n* = 6:7. (g) Representative images of Picrosirius red staining of mouse cardiac sections detecting interstitial and perivascular fibrosis. Scale bar: 100 μm. (h, i) Quantifications of interstitial fibrosis area/total area and perivascular fibrosis area/lumen area. *n* = 6:7. Values are expressed as mean ± SEM. Data were analyzed using two‐way ANOVA with Tukey's test for multiple comparisons (c, d) or unpaired Student's *t*‐test (f, h, i).

To examine the potential cardioprotective effect of S100a8/a9 inhibitor ABR‐238901 on DOX‐induced cardiotoxicity, we treated mice with ABR‐238901 for three consecutive days after acute DOX administration (Figure 7a). Echocardiographic analyses showed that ABR‐treatment significantly reversed DOX‐induced cardiac dysfunction, resulting in higher EF and FS in the DOX‐ABR group than in the DOX‐Vehicle group (Figure 7b–d). TUNEL staining demonstrated that acute DOX administration‐induced cellular apoptosis was significantly decreased in the DOX‐ABR group (Figure 7e,f). Picrosirius red staining revealed that ABR‐238901 treatment significantly decreased DOX‐induced cardiac interstitial and perivascular fibrosis (Figure 7g–i). These results together demonstrated the effectiveness of S100a8/a9 inhibition by ABR‐238901 in improving DOX‐induced cardiotoxicity.

## 4 DISCUSSION

**FIGURE 8:**
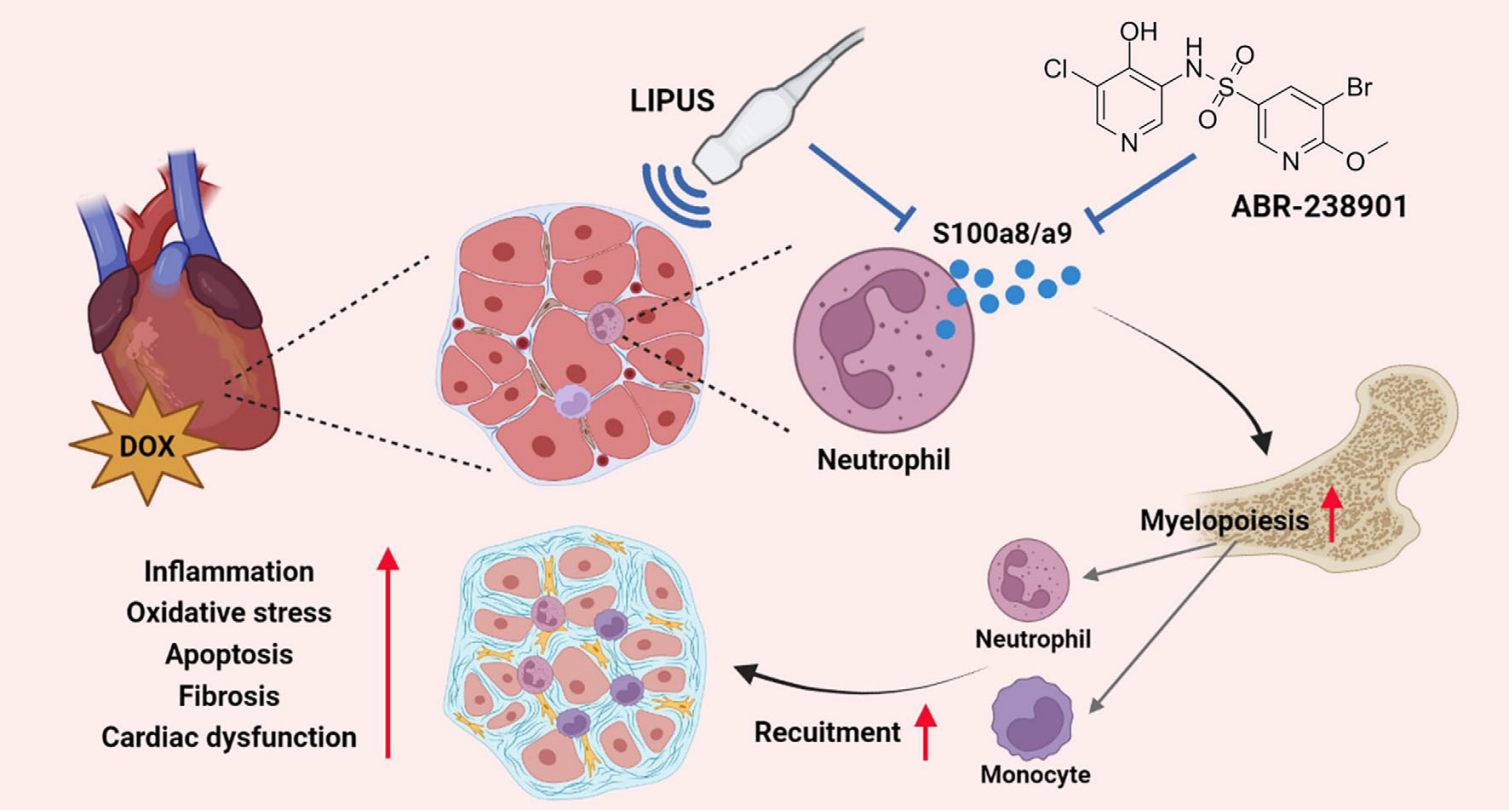
The present study illustrated the beneficial role of LIPUS in preventing DOX‐induced cardiotoxicity in vivo. Mechanistic studies revealed that S100a8/a9‐mediated neutrophil chemotaxis was mitigated by LIPUS treatment, leading to decreased inflammation, oxidative stress, apoptosis, and cardiac fibrosis. ABR‐238901, an S100a8/a9 inhibitor, showed a similar protective effect on DOX‐induced cardiac dysfunction to LIPUS treatment.

The present study discovered that non‐invasive LIPUS treatment protected against DOX‐induced cardiotoxicity, likely through inhibition of S100a8/a9‐mediated neutrophil recruitment and cardiac infiltration. Moreover, inhibition of S100a8/a9 by ABR‐238901 showed a similar protective effect on DOX‐induced cardiotoxicity to LIPUS therapy (Figure 8). To the best of our knowledge, this is the first evidence showing the therapeutic effect of LIPUS on DOX‐induced cardiotoxicity, indicating that LIPUS may potentially become an effective and non‐invasive approach for cardiac dysfunction in patients undergoing DOX chemotherapy.

The high affinity of DOX to cardiomyocytes results in dose‐dependent cardiotoxicity and unreversible dilated cardiomyopathy and heart failure in the end, which remains an important challenge in chemotherapy patients taking DOX.^26^ The progression of DOX‐induced cardiomyopathy involves both local cytotoxicity and systemic immune responses. DOX‐induced cardiotoxicity is mediated by topoisomerase‐IIb in cardiomyocytes, resulting in breaks of DNA double‐strand, defective mitochondrial biogenesis and generation of reactive oxygen species.^27^ Meanwhile, inflammatory cell infiltration in the early phase after DOX administration can be responsible for the later onset of cardiac dysfunction.^28^ Substantial efforts have been made to attenuate or prevent such cardiac side effects. For example, Afrin et al.^29^ have developed a vascular cell adhesion molecule 1‐targeted peptide‐based (VHPKQHRGGSKGC) fluorescent nanoprobe to detect DOX‐induced cardiotoxicity at an early stage, thereby providing a potential strategy for early diagnosis. Furthermore, Chen et al.^30^ have demonstrated that the administration of empagliflozin mitigates DOX‐induced cardiotoxicity by enhancing mitochondrial biogenesis. Similarly, Ma et al.^31^ have reported that the inhalation of hydrogen protects against DOX‐induced cardiotoxicity by stimulating autophagy. However, effective and safe strategies remain limited. As a safe and non‐invasive approach, LIPUS has shown great potential in tissue repair and resolution of inflammation in various diseases.^32^, ^33^, ^34^, ^35^ Of note, the therapeutic effects of LIPUS on cardiovascular diseases have been reported in recent years.^10^, ^11^, ^12^, ^13^, ^14^ In our present study, we showed that LIPUS therapy provided a great benefit in both acute and chronic DOX injury models, including the improvement of cardiac contractile function, the attenuation of myocardial injury and cellular apoptosis, as well as the alleviation of oxidative stress and cardiac fibrosis.

The importance of immunity and inflammatory mechanisms in cardiovascular diseases has drawn more and more attentions.^36^ Strategies to prevent overt immune activation and inflammatory responses have shown great potentials for treating cardiovascular diseases, including DOX‐induced cardiotoxicity.^37^, ^38^ The unbiased RNA‐seq results in our present study revealed that neutrophil chemotaxis inhibition might be involved in the cardioprotective effects of LIPUS therapy. Further flow cytometry analyses illustrated the inhibitory effect of LIPUS treatment on DOX‐induced neutrophil recruitment and infiltration in the heart. DOX‐induced cardiac neutrophil recruitment is preceded by endothelial dysfunction and pro‐inflammatory cytokines secretion.^7^ Pro‐inflammatory cytokines secreted by several cell types in the heart may activate neutrophil degranulation, endothelial inflammation, and neutrophil transmigration to inflammatory sites.^7^, ^39^ The overt neutrophil activation perpetuates acute and chronic cardiovascular diseases, including DOX‐induced cardiotoxicity.^6^, ^40^ Notably, neutrophil depletion by anti‐Ly6G antibody was reported to protect against DOX‐induced cardiac dysfunction in wild‐type mice, which was also found in mice treated with neutrophil recruitment inhibitor SB265610, suggesting that cardiac neutrophil recruitment plays an essential role in DOX‐induced cardiotoxicity.^6^

Our results further indicated that S100a8/a9 could be the key mediator of DOX‐induced neutrophil recruitment to the heart, and inhibition of S100a8/a9‐mediated cardiac neutrophil recruitment might contribute to the protective effect of LIPUS therapy. S100a8 and S100a9 are important members of the S100 family and are also named myeloid‐related protein 8 (MRP8) and MRP14. They preferentially form S100a8/a9 heterodimer complexes due to the poor stability of homodimers. S100a8/a9 is constitutionally expressed in myeloid cells, especially neutrophils. Neutrophil‐derived S100a8/a9 has recently been demonstrated to promote granulopoiesis/myelopoiesis in the bone marrow after myocardial infarction.^25^, ^41^ S100a8/a9 then binds to Toll‐like receptor 4 on resident or recruited neutrophils to prime the Nlrp3 inflammasome and secretion of IL‐1β, which then interacts with its receptor (interleukin 1 receptor type 1) on hematopoietic stem and progenitor cells in the bone marrow to stimulate myelopoiesis in a cell‐intrinsic manner.^25^ Genetic and pharmacological strategies aiming at blockade of the S100a8/a9‐Nlrp3‐IL‐1β signaling axis have been shown to dampen myelopoiesis and improve cardiac function after myocardial infarction.^19^, ^25^, ^42^, ^43^ Of note, S100a8 and S100a9 are also increased in the hearts of DOX‐treated mice,^44^, ^45^ while genetic deletion of S100a8/S100a9 attenuates DOX‐induced cardiac dysfunction.^45^ Our present study confirmed the activation of S100a8/a9‐Nlrp3‐IL‐1β signaling in DOX‐treated hearts, which was dampened by LIPUS therapy. Moreover, S100a8/a9 inhibition by ABR‐238901 decreased DOX‐induced neutrophil accumulation in the heart.

With the development of cancer therapy and the improvement of survival, there is increasing recognition of the short‐ and long‐term complications of cancer treatments that affect morbidity and mortality, including cardiovascular toxicity.^46^, ^47^ The discipline of cardio‐oncology has emerged to better cope with cardiovascular complications in cancer patients.^47^ The beneficial effects of non‐invasive LIPUS therapy on DOX‐induced cardiotoxicity demonstrated great potentials in treating cardiac events in cancer patients undergoing chemotherapy. Furthermore, inhibition of S100a8/a9 by ABR‐238901 in our present study showed a similar cardioprotective effect to LIPUS treatment. As a potent inhibitor of S100a8/a9, ABR‐238901 treatment has been shown to reduce tumor load in combination with Bortezomib in experimental multiple myeloma.^48^ More importantly, short‐term ABR‐238901 treatment post‐myocardial infarction has been shown to inhibit inflammation and improve cardiac function,^18^, ^19^ indicating the potential of S100a8/a9 inhibitor as an immunomodulatory treatment of cardiotoxicity in cancer patients undergoing chemotherapy.

Our present study also has some limitations. First, the parameters of LIPUS treatment were referred to our previous experiences, which might not be the most efficient in alleviating DOX‐induced cardiotoxicity. Second, although we focused on the inhibition of S100a8/a9‐mediated neutrophil recruitment as a main mechanism of the LIPUS therapy, other mechanisms may also be involved. Third, the exact cell type activated by LIPUS therapy and the mechanotransduction pathway involved remain unknown and warrant further investigation. Fourth, in light of the greater susceptibility of male mice^49^ and human beings^50^ to DOX‐induced cardiotoxicity, we exclusively used male mice in all animal experiments, thereby potentially limiting the generalizability of our findings in the female population.

## 5 CONCLUSIONS

Collectively, we demonstrated that LIPUS therapy elicited a protective effect on cardiac dysfunction and myocardial injury induced by DOX at least partially through S100a8/a9‐mediated cardiac neutrophil recruitment. ABR‐238901, a potent S100a8/a9 inhibitor, showed a similar effect on DOX‐induced cardiac dysfunction to LIPUS treatment. LIPUS may be a promising and non‐invasive treatment strategy for DOX‐induced cardiotoxicity.

## AUTHOR CONTRIBUTIONS

**Hong Zhu:** Conceptualization (lead); data curation (lead); formal analysis (lead); investigation (lead); methodology (lead); project administration (lead); validation (lead); visualization (lead); writing – original draft (lead); writing – review and editing (lead). **Min He:** Conceptualization (equal); data curation (equal); formal analysis (equal); investigation (equal); methodology (equal); project administration (equal); validation (equal); visualization (equal). **Yong‐Li Wang:** Conceptualization (equal); data curation (equal); formal analysis (supporting); investigation (supporting); methodology (supporting); project administration (supporting); writing – review and editing (supporting). **Yuanxin Zhang:** Data curation (supporting); formal analysis (supporting); investigation (supporting); methodology (supporting); writing – review and editing (supporting). **Jingsong Dong:** Investigation (supporting); methodology (supporting); validation (supporting). **Bo‐Yan Chen:** Methodology (supporting); validation (supporting); writing – review and editing (supporting). **Yu‐Lin Li:** Methodology (supporting); writing – review and editing (supporting). **Lu‐Jun Zhou:** Methodology (supporting); validation (supporting). **Lin‐Juan Du:** Investigation (supporting); methodology (supporting); writing – review and editing (supporting). **Yuan Liu:** Methodology (supporting); writing – review and editing (supporting). **Wu‐Chang Zhang:** Project administration (supporting); supervision (supporting); writing – review and editing (supporting). **Dean Ta:** Conceptualization (equal); funding acquisition (equal); supervision (equal); validation (equal). **Sheng‐Zhong Duan:** Conceptualization (equal); funding acquisition (lead); supervision (lead); validation (equal); writing – review and editing (equal).

## CONFLICT OF INTEREST STATEMENT

The authors have declared that no conflict of interest exists.

## Supporting information


**Data S1:** Supporting Inforamtion.

## Data Availability

The data that support the findings of this study are available from the corresponding author upon reasonable request.
