# Supplementary material for: Low‐intensity pulsed ultrasound alleviates doxorubicin‐induced cardiotoxicity via inhibition of S100a8/a9‐mediated cardiac recruitment of neutrophils
Source: Bioeng Transl Med. 2023 Jul 7;8(6):e10570. doi: 10.1002/btm2.10570 (PMC10658545; doi:10.1002/btm2.10570)
Supplement: Supplementary file 1 — Data S1: Supporting Inforamtion. [file BTM2-8-e10570-s001.docx]

**Supplemental tables**

**Table S1. Primer sequence information**

| **Gene name** | **Forward (5’-3’)** | **Reverse (5’-3’)** |
| --- | --- | --- |
| *S100a8* | AAATCACCATGCCCTCTACAAG | CCCACTTTTATCACCATCGCAA |
| *S100a9* | ATACTCTAGGAAGGAAGGACACC | TCCATGATGTCATTTATGAGGGC |
| *Csf3r* | CTGATCTTCTTGCTACTCCCCA | GGTGTAGTTCAAGTGAGGCAG |
| *Ccl22* | AGGTCCCTATGGTGCCAATGT | CGGCAGGATTTTGAGGTCCA |
| *Cxcl2* | CCAACCACCAGGCTACAGG | GCGTCACACTCAAGCTCTG |
| *Ccl2* | TTAAAAACCTGGATCGGAACCAA | GCATTAGCTTCAGATTTACGGGT |
| *Il1b* | GCAACTGTTCCTGAACTCAACT | ATCTTTTGGGGTCCGTCAACT |
| *Nlrp3* | ATTACCCGCCCGAGAAAGG | TCGCAGCAAAGATCCACACAG |
| *Gapdh* | AGGTCGGTGTGAACGGATTTG | TGTAGACCATGTAGTTGAGGTCA |

**Supplemental figures and figure legends**


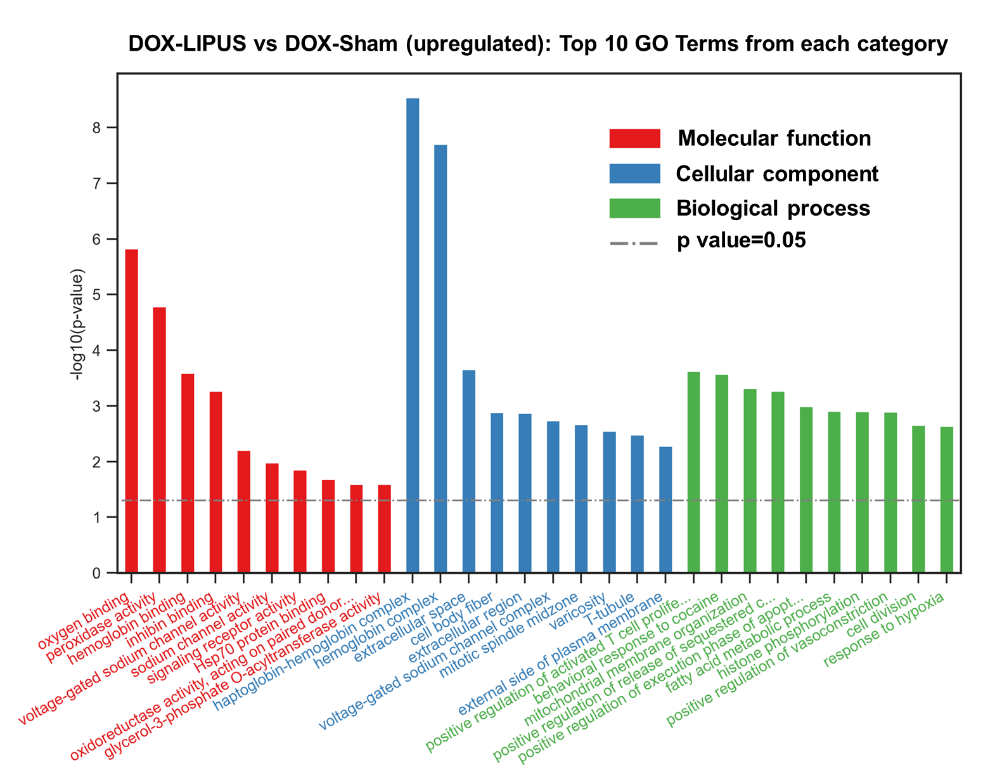


**Fig S1. GO analysis of upregulated genes after LIPUS treatment.**

GO analysis of RNA-seq data showing the top 10 terms from each of the 3 categories (molecular function, cellular component, and biological process) based on the upregulated DEGs between DOX-LIPUS and DOX-Sham.


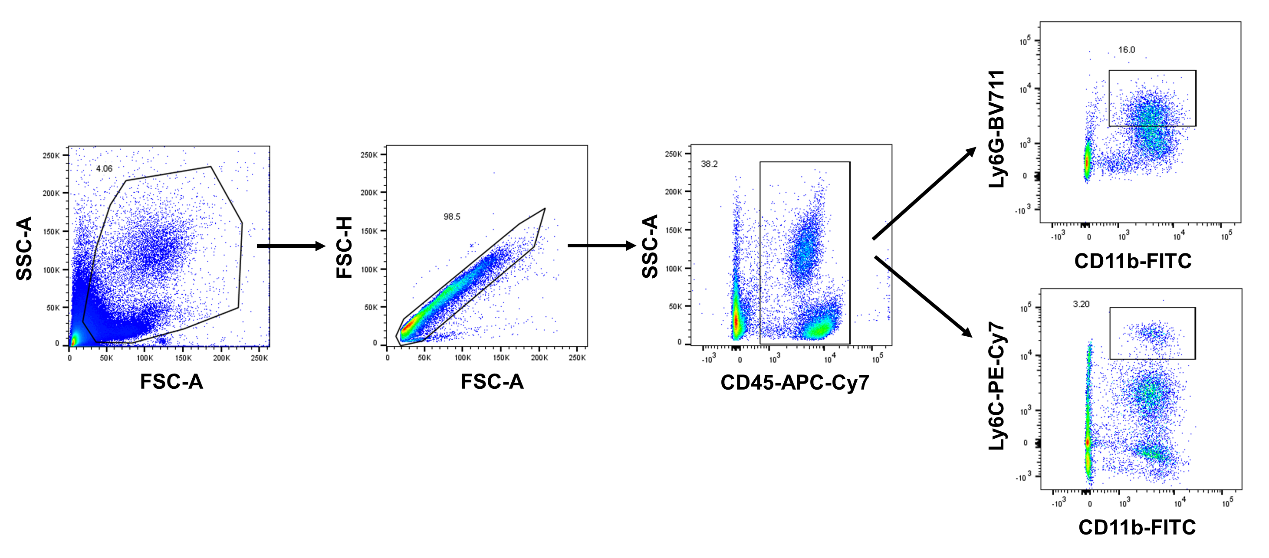


**Fig S2. Gating strategy for flow cytometric analysis.**

Single-cell suspension of heart samples or blood were prepared and labeled with various antibodies. Doublets were excluded and CD45 was used to identity CD45^+^ immune cells, which included neutrophils and monocytes. Neutrophils were selected by CD11b^+^Ly6G^+^ and monocytes were stratified as CD11b^+^Ly6C^+^.


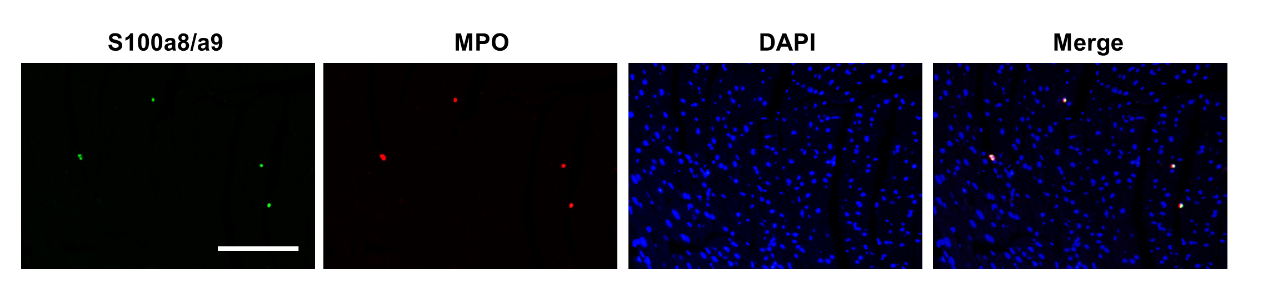


**Fig S3. Double immunofluorescence staining of S100a8/a9 and MPO in the heart after DOX administration.**

Representative immunofluorescence staining of S100a8/a9 (green) and MPO (red) in cardiac sections. Nuclei were stained in blue with DAPI. Scale bar: 200 μm.
